# (4-{[(Pyridin-4-yl)methyl­idene]amino}­phen­yl)ferrocene

**DOI:** 10.1107/S1600536812047812

**Published:** 2012-11-24

**Authors:** Vincent O. Nyamori, Godfrey Keru, Bernard Omondi

**Affiliations:** a School of Chemistry and Physics, University of KwaZulu-Natal, Westville Campus, Private Bag X54001, Durban 4000, South Africa

## Abstract

The asymetric unit of the title compound, [Fe(C_5_H_5_)(C_17_H_13_N_2_)], contains two independent mol­ecules whose conformations differ, especially in the 4-{(pyridin-4-yl)methyl­idene]amino}­phenyl unit where one is flipped by almost 180°. The cyclo­penta­dienyl rings of the ferrocene unit also exhibit different staggered conformations: in one mol­ecule the conformation is staggered by 9.43 (2)° and in the other by 24.46 (1)° from an ideal eclipsed geometry. The plane of the benzene ring is tilted away from the ferrocene group in both mol­ecules, with dihedral angles of 6.97 (1) and 10.30 (2)°. The benzene ring is also slightly twisted from the plane of the pyridine ring, with dihedral angles of 5.98 (2) and 6.51 (2)° in the two mol­ecules.

## Related literature

For related compounds, see: Nyamori *et al.* (2012 ▶); Nyamori & Moodley (2011 ▶). For the synthesis, see: Rajput *et al.* (2006 ▶).
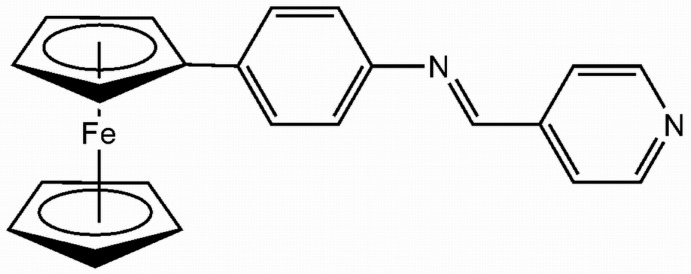


## Experimental

### 

#### Crystal data

[Fe(C_5_H_5_)(C_17_H_13_N_2_)]
*M* *_r_* = 366.23
Monoclinic, 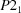

*a* = 10.7200 (3) Å
*b* = 7.4015 (2) Å
*c* = 20.8517 (6) Å
β = 93.044 (1)°
*V* = 1652.12 (8) Å^3^
*Z* = 4
Mo *K*α radiation
μ = 0.92 mm^−1^
*T* = 173 K
0.56 × 0.45 × 0.10 mm

#### Data collection

Bruker SMART APEXII CCD diffractometer
Absorption correction: multi-scan (*SADABS*; Bruker, 2008 ▶) *T*_min_ = 0.627, *T*_max_ = 0.914
36615 measured reflections
7976 independent reflections
7617 reflections with *I* > 2σ(*I*)
*R*_int_ = 0.021

#### Refinement

*R*[*F* ^2^ > 2σ(*F* ^2^)] = 0.022
*wR*(*F* ^2^) = 0.061
*S* = 1.02
7976 reflections
451 parameters
1 restraint
H-atom parameters constrained
Δρ_max_ = 0.35 e Å^−3^
Δρ_min_ = −0.26 e Å^−3^
Absolute structure: Flack (1983 ▶), 3568 Friedel pairs
Flack parameter: 0.003 (8)

### 

Data collection: *APEX2* (Bruker, 2008 ▶); cell refinement: *SAINT-Plus* (Bruker, 2008 ▶); data reduction: *SAINT-Plus* and *XPREP* (Bruker, 2008 ▶); program(s) used to solve structure: *SHELXS97* (Sheldrick, 2008 ▶); program(s) used to refine structure: *SHELXL97* (Sheldrick, 2008 ▶); molecular graphics: *ORTEP-3* (Farrugia, 2012 ▶); software used to prepare material for publication: *WinGX* (Farrugia, 2012 ▶).

## Supplementary Material

Crystal structure: contains datablock(s) global, I. DOI: 10.1107/S1600536812047812/hg5267sup1.cif

Structure factors: contains datablock(s) I. DOI: 10.1107/S1600536812047812/hg5267Isup2.hkl

Additional supplementary materials:  crystallographic information; 3D view; checkCIF report

## Figures and Tables

**Fig. 1.**
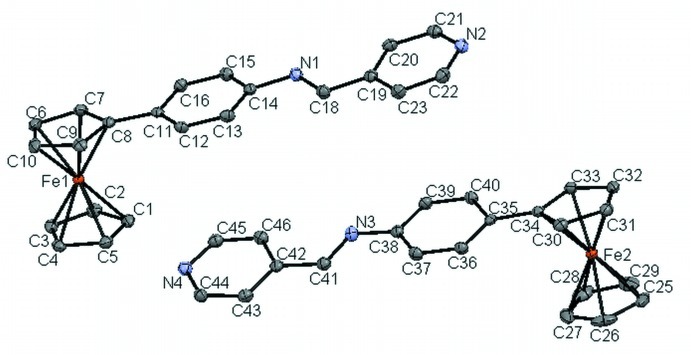
The asymmetric unit of the title compound with displacement ellipsoids drawn at the 50% probability level.

## supplementary crystallographic information

### Comment

As part of investigation of the title compound (I) as a catalyst for synthesis of nitrogen doped shaped carbon nanomaterials we have determined its crystal structure. The compound crystallized with two independent molecules in the asymmetric unit (Fig. 1). In each molecule, the ferrocenyl moiety is linked to phenyl with imine and pyridine groups.

The conformations of the two molecules are different especially in the staggering angles of each ones cyclopentadienyl ring and also in the (pyridin-4-ylmethylene)aniline moieties. The two cyclopentadienyl rings of the ferrocene moiety also exhibit different staggered geometries where in one the conformation is skewed by 9.43 (2)° and in the other by 24.46 (1)°. This differs from the value of 15.9° observed by Nyamori & Moodley, (2011) and 2.17 (2)° by Nyamori *et al.*, (2012) in related structures. The phenyl group is tilted away from the ferrocene group in one of the molecules by a dihedral angle of 6.97 (1)° and by a dihedral angle of 10.30 (2)° in the other molecule. The phenyl ring is also slightly twisted from the plane of the pyridyl ring by a dihedral angle of 5.98 (2)° in one and by 6.51 (2)° in the other molecule.

### Experimental

The title compound was prepared by a modification of the reported method (Rajput *et al.*, 2006). Into a Pyrex tube fitted with a ground glass joint 4-ferrocenylaniline (130 mg,0.47 mmol) and pyridine-4-carboxyaldehyde (76 mg, 0.71 mmol) were added. The compounds were thoroughly ground. The mixture turned into a red solid which was subjected to column chromatography using a mixture of hexane: dichloromethane in the ratio 8:2. (162 mg, 94%) m.p. 199–201°C. IR (ATR cm^-1^) 3430, 3043, 2954, 2876, 1621, 1582, 1465, 1336, 1168, 1083, 1032, 889, 845, 741, 643, 549, 524, 491 δ^1^H (CDCl_3_) = 8.80 (2*H*, dd, J = 4.5 and 1.5 pyH), 8.54 (1*H*, s, CHN), 7.80 (2*H*, dd, J = 4.5 and 1.5 pyH), 7.54(2*H*, dd, J = 6.7 and 1.8 ArH), 4.69 (2*H*, t, J = 1.8 C_5_H_4_), 4.32 (2*H*, t, J = 1.8 C_5_H_4_), 4.06 (5*H*, s, C_5_H_4_). δ^13^C (CDCl_3_) = 151.2, 148.5, 143.5, 138.9, 127.2, 122.9, 121.6, 96.5, 85.4, 77.1, 69.4, 66.4. m/*z*(%) 277.1 (23.9%) 367.2 (100%) Found: *M*+,367.2 for C_22_H_18_FeN_2_, requires *M*, 366.2367.

### Refinement

Carbon-bound H-atoms were placed in calculated positions [C—H = 1.00 for methine H atoms and 0.95 Å for aromatic H atoms; *U*_iso_(H) = 1.2*U*_eq_(C) and were included in the refinement in the riding model approximation

### Crystal data

**Table:**

[Fe(C_5_H_5_)(C_17_H_13_N_2_)]	*F*(000) = 760
*M**_r_* = 366.23	*D*_x_ = 1.472 Mg m^−^^3^
Monoclinic, *P*2_1_	Mo *K*α radiation, λ = 0.71073 Å
Hall symbol: P 2yb	Cell parameters from 36615 reflections
*a* = 10.7200 (3) Å	θ = 1.9–28.3°
*b* = 7.4015 (2) Å	µ = 0.92 mm^−^^1^
*c* = 20.8517 (6) Å	*T* = 173 K
β = 93.044 (1)°	Plate, red
*V* = 1652.12 (8) Å^3^	0.56 × 0.45 × 0.1 mm
*Z* = 4	

### Data collection

**Table:**

Bruker SMART APEXII CCD diffractometer	7617 reflections with *I* > 2σ(*I*)
Graphite monochromator	*R*_int_ = 0.021
φ and ω scans	θ_max_ = 28.3°, θ_min_ = 1.9°
Absorption correction: multi-scan (*SADABS*; Bruker, 2008)	*h* = −14→14
*T*_min_ = 0.627, *T*_max_ = 0.914	*k* = −9→9
36615 measured reflections	*l* = −27→27
7976 independent reflections	

### Refinement

**Table:**

Refinement on *F*^2^	Secondary atom site location: difference Fourier map
Least-squares matrix: full	Hydrogen site location: inferred from neighbouring sites
*R*[*F*^2^ > 2σ(*F*^2^)] = 0.022	H-atom parameters constrained
*wR*(*F*^2^) = 0.061	*w* = 1/[σ^2^(*F*_o_^2^) + (0.0387*P*)^2^ + 0.2134*P*] where *P* = (*F*_o_^2^ + 2*F*_c_^2^)/3
*S* = 1.02	(Δ/σ)_max_ = 0.004
7976 reflections	Δρ_max_ = 0.35 e Å^−^^3^
451 parameters	Δρ_min_ = −0.26 e Å^−^^3^
1 restraint	Absolute structure: Flack (1983), 3568 Friedel pairs
Primary atom site location: structure-invariant direct methods	Flack parameter: 0.003 (8)

### Special details

**Table:**

Geometry. All e.s.d.'s (except the e.s.d. in the dihedral angle between two l.s. planes) are estimated using the full covariance matrix. The cell e.s.d.'s are taken into account individually in the estimation of e.s.d.'s in distances, angles and torsion angles; correlations between e.s.d.'s in cell parameters are only used when they are defined by crystal symmetry. An approximate (isotropic) treatment of cell e.s.d.'s is used for estimating e.s.d.'s involving l.s. planes.
Refinement. Refinement of *F*^2^ against ALL reflections. The weighted *R*-factor *wR* and goodness of fit *S* are based on *F*^2^, conventional *R*-factors *R* are based on *F*, with *F* set to zero for negative *F*^2^. The threshold expression of *F*^2^ > σ(*F*^2^) is used only for calculating *R*-factors(gt) *etc*. and is not relevant to the choice of reflections for refinement. *R*-factors based on *F*^2^ are statistically about twice as large as those based on *F*, and *R*- factors based on ALL data will be even larger.

### Fractional atomic coordinates and isotropic or equivalent isotropic displacement parameters (Å^2^)

**Table:**

	*x*	*y*	*z*	*U*_iso_*/*U*_eq_	
C1	0.20957 (14)	0.3249 (2)	0.67558 (7)	0.0191 (3)	
H1	0.1816	0.3573	0.7191	0.023*	
C2	0.15953 (15)	0.3946 (2)	0.61587 (7)	0.0185 (3)	
H2	0.0908	0.4855	0.6102	0.022*	
C3	0.22454 (14)	0.3131 (2)	0.56577 (7)	0.0171 (3)	
H3	0.2098	0.3369	0.5188	0.021*	
C4	0.31388 (13)	0.1905 (2)	0.59426 (7)	0.0166 (3)	
H4	0.3729	0.1133	0.5707	0.02*	
C5	0.30458 (13)	0.1976 (2)	0.66213 (7)	0.0178 (3)	
H5	0.3554	0.1255	0.6946	0.021*	
C6	0.00589 (14)	0.0038 (3)	0.55626 (7)	0.0211 (3)	
H6	−0.0177	0.0449	0.5116	0.025*	
C7	−0.04881 (13)	0.0643 (2)	0.61311 (7)	0.0179 (3)	
H7	−0.1176	0.155	0.6152	0.022*	
C8	0.01177 (13)	−0.0265 (2)	0.66683 (7)	0.0149 (3)	
C9	0.10583 (14)	−0.1422 (2)	0.64227 (8)	0.0174 (3)	
H9	0.1644	−0.2215	0.6684	0.021*	
C10	0.10134 (15)	−0.1233 (2)	0.57398 (7)	0.0199 (3)	
H10	0.1563	−0.187	0.544	0.024*	
C11	−0.01936 (12)	−0.0109 (2)	0.73452 (6)	0.0139 (3)	
C12	0.04916 (13)	−0.1044 (2)	0.78316 (7)	0.0158 (3)	
H12	0.1193	−0.1745	0.7724	0.019*	
C13	0.01676 (13)	−0.0967 (2)	0.84662 (7)	0.0169 (3)	
H13	0.0647	−0.1607	0.8788	0.02*	
C14	−0.08678 (12)	0.0055 (2)	0.86320 (6)	0.0145 (3)	
C15	−0.15556 (12)	0.0982 (2)	0.81531 (7)	0.0162 (3)	
H15	−0.2261	0.1674	0.8261	0.019*	
C16	−0.12222 (12)	0.0908 (2)	0.75184 (7)	0.0159 (3)	
H16	−0.17	0.1556	0.7198	0.019*	
C18	−0.06579 (13)	−0.0307 (2)	0.97509 (7)	0.0182 (3)	
H18	0.0152	−0.079	0.9701	0.022*	
C19	−0.11390 (13)	−0.0168 (2)	1.03980 (7)	0.0160 (3)	
C20	−0.22562 (13)	0.0726 (2)	1.05145 (7)	0.0171 (3)	
H20	−0.2736	0.1285	1.0174	0.021*	
C21	−0.26468 (13)	0.0777 (2)	1.11383 (7)	0.0181 (3)	
H21	−0.3403	0.1391	1.1214	0.022*	
C22	−0.09449 (14)	−0.0818 (2)	1.15239 (7)	0.0195 (3)	
H22	−0.048	−0.1356	1.1874	0.023*	
C23	−0.04700 (14)	−0.0934 (2)	1.09183 (7)	0.0183 (3)	
H23	0.0301	−0.153	1.0861	0.022*	
C25	0.47353 (17)	0.2943 (3)	1.44743 (8)	0.0317 (5)	
H25	0.4737	0.2766	1.495	0.038*	
C26	0.55420 (15)	0.2065 (3)	1.40464 (10)	0.0325 (4)	
H26	0.6216	0.1173	1.4165	0.039*	
C27	0.52195 (17)	0.2742 (3)	1.34222 (8)	0.0308 (4)	
H27	0.5618	0.2389	1.3018	0.037*	
C28	0.42534 (18)	0.3972 (3)	1.34697 (9)	0.0321 (4)	
H28	0.3834	0.4646	1.3103	0.039*	
C29	0.39537 (16)	0.4108 (3)	1.41095 (10)	0.0314 (4)	
H29	0.329	0.4898	1.4279	0.038*	
C30	0.35572 (14)	−0.1180 (2)	1.35855 (7)	0.0164 (3)	
H30	0.4263	−0.2022	1.3508	0.02*	
C31	0.30509 (14)	−0.0828 (2)	1.41874 (7)	0.0182 (3)	
H31	0.3344	−0.1371	1.4608	0.022*	
C32	0.20671 (13)	0.0452 (2)	1.40931 (7)	0.0182 (3)	
H32	0.155	0.0964	1.4435	0.022*	
C33	0.19597 (13)	0.0893 (2)	1.34280 (7)	0.0159 (3)	
H33	0.1352	0.1768	1.3221	0.019*	
C34	0.28865 (12)	−0.0117 (2)	1.31064 (7)	0.0138 (3)	
C35	0.30847 (12)	−0.0040 (2)	1.24143 (6)	0.0137 (3)	
C36	0.41133 (13)	−0.0874 (2)	1.21457 (7)	0.0160 (3)	
H36	0.4725	−0.1457	1.242	0.019*	
C37	0.42527 (13)	−0.0863 (2)	1.14895 (7)	0.0171 (3)	
H37	0.4952	−0.1444	1.1318	0.021*	
C38	0.33697 (13)	0.0000 (2)	1.10776 (7)	0.0154 (3)	
C39	0.23483 (13)	0.0843 (2)	1.13411 (7)	0.0162 (3)	
H39	0.1741	0.1434	1.1067	0.019*	
C40	0.22120 (12)	0.0825 (2)	1.19986 (7)	0.0155 (3)	
H40	0.1514	0.1411	1.2169	0.019*	
C41	0.43585 (13)	−0.0300 (2)	1.01043 (7)	0.0176 (3)	
H41	0.5087	−0.0708	1.034	0.021*	
C42	0.43572 (13)	−0.0157 (2)	0.94004 (7)	0.0157 (3)	
C43	0.53776 (14)	−0.0773 (2)	0.90737 (7)	0.0177 (3)	
H43	0.6098	−0.1232	0.9304	0.021*	
C44	0.53226 (14)	−0.0705 (2)	0.84080 (7)	0.0185 (3)	
H44	0.6012	−0.1168	0.8191	0.022*	
C45	0.33986 (14)	0.0620 (2)	0.83717 (7)	0.0185 (3)	
H45	0.2716	0.1143	0.8129	0.022*	
C46	0.33457 (13)	0.0570 (2)	0.90332 (7)	0.0176 (3)	
H46	0.2636	0.1022	0.9235	0.021*	
N1	−0.13098 (11)	0.02096 (18)	0.92604 (6)	0.0166 (2)	
N2	−0.20154 (12)	0.00054 (19)	1.16425 (6)	0.0191 (3)	
N3	0.33899 (11)	0.01199 (18)	1.03994 (6)	0.0176 (3)	
N4	0.43575 (11)	−0.00276 (19)	0.80527 (6)	0.0187 (3)	
Fe1	0.137820 (17)	0.12015 (2)	0.617436 (8)	0.01160 (6)	
Fe2	0.371923 (17)	0.14988 (2)	1.379891 (9)	0.01317 (6)	

### Atomic displacement parameters (Å^2^)

**Table:**

	*U*^11^	*U*^22^	*U*^33^	*U*^12^	*U*^13^	*U*^23^
C1	0.0227 (7)	0.0168 (8)	0.0180 (7)	−0.0065 (6)	0.0043 (6)	−0.0047 (6)
C2	0.0210 (7)	0.0111 (8)	0.0234 (8)	−0.0012 (6)	0.0010 (6)	−0.0004 (6)
C3	0.0228 (7)	0.0144 (8)	0.0141 (7)	−0.0048 (6)	0.0008 (6)	0.0020 (6)
C4	0.0146 (6)	0.0157 (8)	0.0200 (7)	−0.0028 (5)	0.0048 (5)	−0.0005 (6)
C5	0.0163 (7)	0.0193 (9)	0.0174 (7)	−0.0041 (6)	−0.0019 (5)	0.0020 (6)
C6	0.0211 (7)	0.0266 (9)	0.0154 (7)	−0.0099 (6)	−0.0019 (6)	−0.0001 (6)
C7	0.0140 (6)	0.0216 (9)	0.0179 (7)	−0.0036 (5)	−0.0008 (5)	0.0014 (6)
C8	0.0159 (6)	0.0130 (8)	0.0158 (6)	−0.0046 (5)	0.0006 (5)	−0.0004 (6)
C9	0.0212 (7)	0.0100 (8)	0.0213 (7)	−0.0023 (6)	0.0035 (6)	−0.0013 (6)
C10	0.0254 (8)	0.0166 (8)	0.0183 (7)	−0.0062 (6)	0.0051 (6)	−0.0062 (6)
C11	0.0137 (6)	0.0127 (7)	0.0153 (6)	−0.0046 (5)	0.0013 (5)	0.0002 (5)
C12	0.0129 (6)	0.0155 (8)	0.0190 (7)	0.0010 (5)	0.0014 (5)	0.0012 (6)
C13	0.0140 (6)	0.0168 (8)	0.0196 (7)	0.0008 (5)	−0.0007 (5)	0.0034 (6)
C14	0.0147 (6)	0.0136 (7)	0.0154 (6)	−0.0026 (5)	0.0016 (5)	−0.0007 (5)
C15	0.0153 (6)	0.0141 (7)	0.0194 (7)	0.0009 (5)	0.0018 (5)	−0.0012 (6)
C16	0.0165 (6)	0.0151 (8)	0.0158 (6)	0.0012 (5)	−0.0017 (5)	0.0013 (5)
C18	0.0168 (7)	0.0183 (8)	0.0195 (7)	0.0015 (6)	0.0026 (5)	−0.0010 (6)
C19	0.0182 (7)	0.0137 (7)	0.0160 (6)	−0.0026 (5)	0.0005 (5)	−0.0001 (5)
C20	0.0161 (6)	0.0164 (8)	0.0185 (7)	−0.0012 (5)	−0.0020 (5)	0.0019 (5)
C21	0.0166 (7)	0.0161 (8)	0.0217 (7)	−0.0008 (5)	0.0019 (5)	0.0005 (6)
C22	0.0226 (7)	0.0178 (9)	0.0177 (7)	−0.0017 (6)	−0.0022 (6)	0.0036 (6)
C23	0.0178 (7)	0.0169 (8)	0.0202 (7)	0.0024 (6)	0.0003 (5)	0.0000 (6)
C25	0.0372 (9)	0.0420 (13)	0.0158 (8)	−0.0270 (9)	0.0006 (7)	−0.0029 (7)
C26	0.0161 (7)	0.0211 (10)	0.0593 (12)	−0.0064 (7)	−0.0089 (7)	0.0061 (9)
C27	0.0354 (9)	0.0317 (11)	0.0265 (9)	−0.0191 (8)	0.0149 (7)	−0.0102 (8)
C28	0.0376 (10)	0.0210 (10)	0.0365 (10)	−0.0138 (8)	−0.0092 (8)	0.0079 (8)
C29	0.0268 (8)	0.0176 (9)	0.0505 (12)	−0.0084 (7)	0.0075 (8)	−0.0118 (8)
C30	0.0183 (7)	0.0118 (8)	0.0188 (7)	−0.0014 (5)	−0.0005 (5)	0.0012 (6)
C31	0.0204 (7)	0.0185 (9)	0.0155 (7)	−0.0054 (6)	−0.0006 (5)	0.0021 (6)
C32	0.0173 (7)	0.0192 (8)	0.0183 (7)	−0.0046 (6)	0.0037 (5)	−0.0049 (6)
C33	0.0133 (6)	0.0164 (9)	0.0179 (7)	−0.0002 (5)	0.0009 (5)	−0.0020 (6)
C34	0.0127 (6)	0.0124 (7)	0.0162 (7)	−0.0020 (5)	−0.0001 (5)	−0.0017 (6)
C35	0.0138 (6)	0.0118 (7)	0.0153 (6)	−0.0026 (5)	0.0001 (5)	−0.0025 (5)
C36	0.0148 (6)	0.0156 (8)	0.0176 (7)	0.0008 (5)	0.0008 (5)	−0.0001 (5)
C37	0.0154 (7)	0.0153 (8)	0.0208 (7)	0.0019 (5)	0.0028 (5)	−0.0006 (6)
C38	0.0174 (6)	0.0135 (7)	0.0153 (6)	−0.0019 (5)	0.0015 (5)	−0.0004 (5)
C39	0.0156 (6)	0.0164 (8)	0.0165 (6)	0.0014 (5)	−0.0017 (5)	0.0009 (5)
C40	0.0142 (6)	0.0138 (7)	0.0184 (6)	0.0015 (5)	0.0013 (5)	−0.0010 (5)
C41	0.0180 (7)	0.0168 (8)	0.0179 (7)	−0.0014 (5)	0.0006 (5)	−0.0006 (6)
C42	0.0176 (6)	0.0130 (7)	0.0166 (6)	−0.0034 (5)	0.0009 (5)	−0.0009 (5)
C43	0.0169 (7)	0.0163 (8)	0.0197 (7)	0.0007 (5)	−0.0005 (5)	0.0005 (6)
C44	0.0185 (7)	0.0188 (9)	0.0187 (7)	0.0005 (6)	0.0042 (6)	−0.0013 (6)
C45	0.0178 (7)	0.0167 (8)	0.0209 (7)	−0.0004 (5)	−0.0008 (5)	0.0005 (6)
C46	0.0173 (6)	0.0152 (8)	0.0205 (7)	0.0008 (5)	0.0027 (5)	0.0001 (6)
N1	0.0182 (6)	0.0161 (7)	0.0156 (6)	−0.0017 (5)	0.0029 (4)	0.0000 (5)
N2	0.0212 (6)	0.0187 (7)	0.0176 (6)	−0.0031 (5)	0.0029 (5)	0.0006 (5)
N3	0.0211 (6)	0.0164 (7)	0.0154 (6)	0.0010 (5)	0.0018 (5)	0.0002 (5)
N4	0.0209 (6)	0.0179 (7)	0.0175 (6)	−0.0012 (5)	0.0014 (5)	−0.0009 (5)
Fe1	0.01265 (9)	0.01060 (13)	0.01159 (9)	−0.00095 (7)	0.00106 (7)	−0.00014 (8)
Fe2	0.01361 (9)	0.01336 (14)	0.01255 (9)	−0.00240 (8)	0.00063 (7)	−0.00082 (8)

### Geometric parameters (Å, º)

**Table:**

C1—C2	1.426 (2)	C25—C29	1.399 (3)
C1—C5	1.427 (2)	C25—C26	1.431 (3)
C1—Fe1	2.0638 (16)	C25—Fe2	2.0369 (17)
C1—H1	1	C25—H25	1
C2—C3	1.421 (2)	C26—C27	1.420 (3)
C2—Fe1	2.0450 (17)	C26—Fe2	2.0378 (16)
C2—H2	1	C26—H26	1
C3—C4	1.426 (2)	C27—C28	1.387 (3)
C3—Fe1	2.0423 (15)	C27—Fe2	2.0454 (16)
C3—H3	1	C27—H27	1
C4—C5	1.425 (2)	C28—C29	1.392 (3)
C4—Fe1	2.0404 (14)	C28—Fe2	2.0476 (19)
C4—H4	1	C28—H28	1
C5—Fe1	2.0531 (15)	C29—Fe2	2.0478 (19)
C5—H5	1	C29—H29	1
C6—C7	1.422 (2)	C30—C31	1.418 (2)
C6—C10	1.424 (2)	C30—C34	1.434 (2)
C6—Fe1	2.0440 (15)	C30—Fe2	2.0372 (16)
C6—H6	1	C30—H30	1
C7—C8	1.432 (2)	C31—C32	1.423 (2)
C7—Fe1	2.0402 (14)	C31—Fe2	2.0484 (16)
C7—H7	1	C31—H31	1
C8—C9	1.438 (2)	C32—C33	1.423 (2)
C8—C11	1.4720 (19)	C32—Fe2	2.0564 (14)
C8—Fe1	2.0525 (14)	C32—H32	1
C9—C10	1.429 (2)	C33—C34	1.4374 (19)
C9—Fe1	2.0430 (17)	C33—Fe2	2.0500 (14)
C9—H9	1	C33—H33	1
C10—Fe1	2.0453 (17)	C34—C35	1.4709 (19)
C10—H10	1	C34—Fe2	2.0433 (14)
C11—C16	1.3983 (19)	C35—C40	1.3966 (19)
C11—C12	1.403 (2)	C35—C36	1.4060 (19)
C12—C13	1.387 (2)	C36—C37	1.384 (2)
C12—H12	0.95	C36—H36	0.95
C13—C14	1.401 (2)	C37—C38	1.398 (2)
C13—H13	0.95	C37—H37	0.95
C14—C15	1.391 (2)	C38—C39	1.398 (2)
C14—N1	1.4215 (17)	C38—N3	1.4181 (17)
C15—C16	1.3901 (19)	C39—C40	1.3865 (19)
C15—H15	0.95	C39—H39	0.95
C16—H16	0.95	C40—H40	0.95
C18—N1	1.2668 (19)	C41—N3	1.2732 (19)
C18—C19	1.4733 (19)	C41—C42	1.4716 (19)
C18—H18	0.95	C41—H41	0.95
C19—C23	1.390 (2)	C42—C43	1.396 (2)
C19—C20	1.401 (2)	C42—C46	1.401 (2)
C20—C21	1.388 (2)	C43—C44	1.387 (2)
C20—H20	0.95	C43—H43	0.95
C21—N2	1.3467 (19)	C44—N4	1.338 (2)
C21—H21	0.95	C44—H44	0.95
C22—N2	1.334 (2)	C45—N4	1.3421 (19)
C22—C23	1.389 (2)	C45—C46	1.384 (2)
C22—H22	0.95	C45—H45	0.95
C23—H23	0.95	C46—H46	0.95
			
C2—C1—C5	107.87 (14)	Fe2—C32—H32	126
C2—C1—Fe1	68.99 (9)	C32—C33—C34	108.25 (13)
C5—C1—Fe1	69.32 (9)	C32—C33—Fe2	69.96 (8)
C2—C1—H1	126.1	C34—C33—Fe2	69.19 (8)
C5—C1—H1	126.1	C32—C33—H33	125.9
Fe1—C1—H1	126.1	C34—C33—H33	125.9
C3—C2—C1	108.18 (14)	Fe2—C33—H33	125.9
C3—C2—Fe1	69.56 (9)	C30—C34—C33	107.08 (12)
C1—C2—Fe1	70.41 (9)	C30—C34—C35	127.69 (13)
C3—C2—H2	125.9	C33—C34—C35	125.24 (13)
C1—C2—H2	125.9	C30—C34—Fe2	69.19 (8)
Fe1—C2—H2	125.9	C33—C34—Fe2	69.69 (8)
C2—C3—C4	107.98 (13)	C35—C34—Fe2	126.17 (10)
C2—C3—Fe1	69.76 (9)	C40—C35—C36	117.90 (13)
C4—C3—Fe1	69.48 (8)	C40—C35—C34	119.99 (12)
C2—C3—H3	126	C36—C35—C34	122.07 (13)
C4—C3—H3	126	C37—C36—C35	121.27 (13)
Fe1—C3—H3	126	C37—C36—H36	119.4
C5—C4—C3	108.03 (13)	C35—C36—H36	119.4
C5—C4—Fe1	70.11 (8)	C36—C37—C38	120.33 (13)
C3—C4—Fe1	69.63 (8)	C36—C37—H37	119.8
C5—C4—H4	126	C38—C37—H37	119.8
C3—C4—H4	126	C39—C38—C37	118.76 (13)
Fe1—C4—H4	126	C39—C38—N3	114.71 (12)
C4—C5—C1	107.94 (13)	C37—C38—N3	126.53 (13)
C4—C5—Fe1	69.15 (8)	C40—C39—C38	120.70 (13)
C1—C5—Fe1	70.13 (8)	C40—C39—H39	119.7
C4—C5—H5	126	C38—C39—H39	119.7
C1—C5—H5	126	C39—C40—C35	121.04 (13)
Fe1—C5—H5	126	C39—C40—H40	119.5
C7—C6—C10	108.30 (13)	C35—C40—H40	119.5
C7—C6—Fe1	69.48 (8)	N3—C41—C42	120.46 (13)
C10—C6—Fe1	69.67 (9)	N3—C41—H41	119.8
C7—C6—H6	125.8	C42—C41—H41	119.8
C10—C6—H6	125.8	C43—C42—C46	117.56 (13)
Fe1—C6—H6	125.8	C43—C42—C41	120.27 (13)
C6—C7—C8	108.32 (14)	C46—C42—C41	122.16 (13)
C6—C7—Fe1	69.76 (8)	C44—C43—C42	118.93 (14)
C8—C7—Fe1	69.98 (8)	C44—C43—H43	120.5
C6—C7—H7	125.8	C42—C43—H43	120.5
C8—C7—H7	125.8	N4—C44—C43	123.94 (14)
Fe1—C7—H7	125.8	N4—C44—H44	118
C7—C8—C9	107.35 (13)	C43—C44—H44	118
C7—C8—C11	126.64 (14)	N4—C45—C46	123.88 (13)
C9—C8—C11	125.96 (13)	N4—C45—H45	118.1
C7—C8—Fe1	69.06 (8)	C46—C45—H45	118.1
C9—C8—Fe1	69.10 (8)	C45—C46—C42	118.90 (13)
C11—C8—Fe1	128.95 (10)	C45—C46—H46	120.6
C10—C9—C8	108.04 (14)	C42—C46—H46	120.6
C10—C9—Fe1	69.63 (10)	C18—N1—C14	121.56 (12)
C8—C9—Fe1	69.80 (9)	C22—N2—C21	116.78 (12)
C10—C9—H9	126	C41—N3—C38	121.52 (13)
C8—C9—H9	126	C44—N4—C45	116.72 (12)
Fe1—C9—H9	126	C4—Fe1—C7	163.42 (6)
C6—C10—C9	107.99 (14)	C4—Fe1—C3	40.89 (6)
C6—C10—Fe1	69.57 (10)	C7—Fe1—C3	126.24 (6)
C9—C10—Fe1	69.46 (9)	C4—Fe1—C9	118.18 (6)
C6—C10—H10	126	C7—Fe1—C9	68.98 (7)
C9—C10—H10	126	C3—Fe1—C9	152.31 (6)
Fe1—C10—H10	126	C4—Fe1—C6	125.29 (6)
C16—C11—C12	117.94 (12)	C7—Fe1—C6	40.76 (6)
C16—C11—C8	120.79 (12)	C3—Fe1—C6	106.54 (6)
C12—C11—C8	121.20 (13)	C9—Fe1—C6	68.77 (7)
C13—C12—C11	121.44 (13)	C4—Fe1—C10	106.10 (6)
C13—C12—H12	119.3	C7—Fe1—C10	68.77 (6)
C11—C12—H12	119.3	C3—Fe1—C10	117.65 (6)
C12—C13—C14	119.93 (13)	C9—Fe1—C10	40.92 (6)
C12—C13—H13	120	C6—Fe1—C10	40.77 (7)
C14—C13—H13	120	C4—Fe1—C2	68.62 (6)
C15—C14—C13	119.08 (13)	C7—Fe1—C2	108.22 (7)
C15—C14—N1	115.60 (12)	C3—Fe1—C2	40.68 (6)
C13—C14—N1	125.31 (13)	C9—Fe1—C2	165.63 (6)
C16—C15—C14	120.71 (13)	C6—Fe1—C2	118.96 (7)
C16—C15—H15	119.6	C10—Fe1—C2	152.54 (7)
C14—C15—H15	119.6	C4—Fe1—C8	153.59 (6)
C15—C16—C11	120.89 (13)	C7—Fe1—C8	40.96 (6)
C15—C16—H16	119.6	C3—Fe1—C8	164.75 (6)
C11—C16—H16	119.6	C9—Fe1—C8	41.10 (6)
N1—C18—C19	120.89 (13)	C6—Fe1—C8	68.79 (6)
N1—C18—H18	119.6	C10—Fe1—C8	68.96 (6)
C19—C18—H18	119.6	C2—Fe1—C8	127.72 (6)
C23—C19—C20	117.91 (13)	C4—Fe1—C5	40.74 (6)
C23—C19—C18	119.68 (13)	C7—Fe1—C5	154.79 (6)
C20—C19—C18	122.41 (13)	C3—Fe1—C5	68.57 (6)
C21—C20—C19	118.60 (13)	C9—Fe1—C5	107.69 (6)
C21—C20—H20	120.7	C6—Fe1—C5	163.29 (7)
C19—C20—H20	120.7	C10—Fe1—C5	126.03 (7)
N2—C21—C20	123.77 (13)	C2—Fe1—C5	68.47 (6)
N2—C21—H21	118.1	C8—Fe1—C5	120.02 (6)
C20—C21—H21	118.1	C4—Fe1—C1	68.36 (6)
N2—C22—C23	123.87 (14)	C7—Fe1—C1	120.58 (6)
N2—C22—H22	118.1	C3—Fe1—C1	68.31 (6)
C23—C22—H22	118.1	C9—Fe1—C1	127.68 (6)
C22—C23—C19	119.05 (14)	C6—Fe1—C1	154.16 (7)
C22—C23—H23	120.5	C10—Fe1—C1	164.52 (7)
C19—C23—H23	120.5	C2—Fe1—C1	40.60 (6)
C29—C25—C26	107.78 (16)	C8—Fe1—C1	109.17 (6)
C29—C25—Fe2	70.40 (10)	C5—Fe1—C1	40.55 (6)
C26—C25—Fe2	69.47 (10)	C25—Fe2—C30	134.26 (8)
C29—C25—H25	126.1	C25—Fe2—C26	41.13 (8)
C26—C25—H25	126.1	C30—Fe2—C26	108.95 (7)
Fe2—C25—H25	126.1	C25—Fe2—C34	173.31 (7)
C27—C26—C25	106.57 (17)	C30—Fe2—C34	41.16 (6)
C27—C26—Fe2	69.93 (9)	C26—Fe2—C34	132.56 (7)
C25—C26—Fe2	69.40 (9)	C25—Fe2—C27	68.10 (7)
C27—C26—H26	126.7	C30—Fe2—C27	114.42 (7)
C25—C26—H26	126.7	C26—Fe2—C27	40.71 (8)
Fe2—C26—H26	126.7	C34—Fe2—C27	108.35 (6)
C28—C27—C26	108.23 (16)	C25—Fe2—C28	67.29 (8)
C28—C27—Fe2	70.28 (10)	C30—Fe2—C28	144.98 (8)
C26—C27—Fe2	69.36 (9)	C26—Fe2—C28	67.65 (8)
C28—C27—H27	125.9	C34—Fe2—C28	113.94 (7)
C26—C27—H27	125.9	C27—Fe2—C28	39.61 (8)
Fe2—C27—H27	125.9	C25—Fe2—C29	40.04 (8)
C27—C28—C29	109.07 (17)	C30—Fe2—C29	173.85 (7)
C27—C28—Fe2	70.11 (11)	C26—Fe2—C29	68.05 (8)
C29—C28—Fe2	70.13 (11)	C34—Fe2—C29	144.79 (7)
C27—C28—H28	125.5	C27—Fe2—C29	67.14 (7)
C29—C28—H28	125.5	C28—Fe2—C29	39.75 (8)
Fe2—C28—H28	125.5	C25—Fe2—C31	110.67 (7)
C28—C29—C25	108.35 (17)	C30—Fe2—C31	40.61 (6)
C28—C29—Fe2	70.12 (11)	C26—Fe2—C31	115.05 (7)
C25—C29—Fe2	69.55 (11)	C34—Fe2—C31	68.82 (6)
C28—C29—H29	125.8	C27—Fe2—C31	145.95 (8)
C25—C29—H29	125.8	C28—Fe2—C31	173.76 (8)
Fe2—C29—H29	125.8	C29—Fe2—C31	135.00 (7)
C31—C30—C34	108.32 (13)	C25—Fe2—C33	145.40 (7)
C31—C30—Fe2	70.12 (9)	C30—Fe2—C33	68.82 (6)
C34—C30—Fe2	69.65 (9)	C26—Fe2—C33	172.40 (8)
C31—C30—H30	125.8	C34—Fe2—C33	41.12 (5)
C34—C30—H30	125.8	C27—Fe2—C33	132.84 (7)
Fe2—C30—H30	125.8	C28—Fe2—C33	109.70 (7)
C30—C31—C32	108.40 (13)	C29—Fe2—C33	114.87 (7)
C30—C31—Fe2	69.27 (9)	C31—Fe2—C33	68.36 (6)
C32—C31—Fe2	70.01 (9)	C25—Fe2—C32	115.39 (6)
C30—C31—H31	125.8	C30—Fe2—C32	68.52 (6)
C32—C31—H31	125.8	C26—Fe2—C32	146.23 (7)
Fe2—C31—H31	125.8	C34—Fe2—C32	68.87 (6)
C31—C32—C33	107.94 (13)	C27—Fe2—C32	172.39 (8)
C31—C32—Fe2	69.41 (9)	C28—Fe2—C32	134.22 (8)
C33—C32—Fe2	69.48 (8)	C29—Fe2—C32	110.73 (7)
C31—C32—H32	126	C31—Fe2—C32	40.58 (6)
C33—C32—H32	126	C33—Fe2—C32	40.56 (6)
			
C5—C1—C2—C3	−0.94 (17)	C3—C2—Fe1—C4	37.82 (9)
Fe1—C1—C2—C3	−59.52 (11)	C1—C2—Fe1—C4	−81.28 (9)
C5—C1—C2—Fe1	58.58 (11)	C3—C2—Fe1—C7	−124.87 (9)
C1—C2—C3—C4	0.88 (17)	C1—C2—Fe1—C7	116.04 (9)
Fe1—C2—C3—C4	−59.18 (11)	C1—C2—Fe1—C3	−119.09 (13)
C1—C2—C3—Fe1	60.06 (11)	C3—C2—Fe1—C9	158.8 (2)
C2—C3—C4—C5	−0.48 (17)	C1—C2—Fe1—C9	39.7 (3)
Fe1—C3—C4—C5	−59.84 (10)	C3—C2—Fe1—C6	−81.68 (10)
C2—C3—C4—Fe1	59.36 (11)	C1—C2—Fe1—C6	159.22 (9)
C3—C4—C5—C1	−0.10 (17)	C3—C2—Fe1—C10	−46.00 (17)
Fe1—C4—C5—C1	−59.63 (10)	C1—C2—Fe1—C10	−165.10 (12)
C3—C4—C5—Fe1	59.53 (10)	C3—C2—Fe1—C8	−166.25 (8)
C2—C1—C5—C4	0.64 (17)	C1—C2—Fe1—C8	74.66 (11)
Fe1—C1—C5—C4	59.02 (10)	C3—C2—Fe1—C5	81.74 (9)
C2—C1—C5—Fe1	−58.38 (11)	C1—C2—Fe1—C5	−37.35 (9)
C10—C6—C7—C8	−0.54 (18)	C3—C2—Fe1—C1	119.09 (13)
Fe1—C6—C7—C8	−59.57 (11)	C7—C8—Fe1—C4	−165.32 (13)
C10—C6—C7—Fe1	59.03 (11)	C9—C8—Fe1—C4	−46.11 (17)
C6—C7—C8—C9	0.76 (17)	C11—C8—Fe1—C4	73.9 (2)
Fe1—C7—C8—C9	−58.68 (10)	C9—C8—Fe1—C7	119.21 (13)
C6—C7—C8—C11	−176.91 (14)	C11—C8—Fe1—C7	−120.82 (18)
Fe1—C7—C8—C11	123.65 (15)	C7—C8—Fe1—C3	37.2 (3)
C6—C7—C8—Fe1	59.44 (11)	C9—C8—Fe1—C3	156.4 (2)
C7—C8—C9—C10	−0.69 (17)	C11—C8—Fe1—C3	−83.6 (2)
C11—C8—C9—C10	177.00 (14)	C7—C8—Fe1—C9	−119.21 (13)
Fe1—C8—C9—C10	−59.34 (11)	C11—C8—Fe1—C9	119.97 (17)
C7—C8—C9—Fe1	58.65 (10)	C7—C8—Fe1—C6	−37.60 (10)
C11—C8—C9—Fe1	−123.66 (14)	C9—C8—Fe1—C6	81.61 (10)
C7—C6—C10—C9	0.11 (18)	C11—C8—Fe1—C6	−158.43 (16)
Fe1—C6—C10—C9	59.02 (11)	C7—C8—Fe1—C10	−81.45 (10)
C7—C6—C10—Fe1	−58.91 (11)	C9—C8—Fe1—C10	37.76 (9)
C8—C9—C10—C6	0.36 (18)	C11—C8—Fe1—C10	157.73 (15)
Fe1—C9—C10—C6	−59.09 (11)	C7—C8—Fe1—C2	73.27 (11)
C8—C9—C10—Fe1	59.45 (11)	C9—C8—Fe1—C2	−167.51 (9)
C7—C8—C11—C16	4.6 (2)	C11—C8—Fe1—C2	−47.55 (16)
C9—C8—C11—C16	−172.66 (15)	C7—C8—Fe1—C5	158.17 (9)
Fe1—C8—C11—C16	96.22 (16)	C9—C8—Fe1—C5	−82.62 (10)
C7—C8—C11—C12	−178.58 (15)	C11—C8—Fe1—C5	37.35 (16)
C9—C8—C11—C12	4.2 (2)	C7—C8—Fe1—C1	114.92 (10)
Fe1—C8—C11—C12	−86.95 (17)	C9—C8—Fe1—C1	−125.87 (9)
C16—C11—C12—C13	−0.1 (2)	C11—C8—Fe1—C1	−5.91 (15)
C8—C11—C12—C13	−177.04 (14)	C1—C5—Fe1—C4	119.21 (13)
C11—C12—C13—C14	0.3 (2)	C4—C5—Fe1—C7	−169.02 (14)
C12—C13—C14—C15	−0.1 (2)	C1—C5—Fe1—C7	−49.81 (19)
C12—C13—C14—N1	178.58 (14)	C4—C5—Fe1—C3	−37.94 (9)
C13—C14—C15—C16	−0.2 (2)	C1—C5—Fe1—C3	81.27 (10)
N1—C14—C15—C16	−179.05 (13)	C4—C5—Fe1—C9	112.90 (10)
C14—C15—C16—C11	0.4 (2)	C1—C5—Fe1—C9	−127.89 (10)
C12—C11—C16—C15	−0.2 (2)	C4—C5—Fe1—C6	37.9 (3)
C8—C11—C16—C15	176.71 (14)	C1—C5—Fe1—C6	157.1 (2)
N1—C18—C19—C23	172.48 (15)	C4—C5—Fe1—C10	71.38 (11)
N1—C18—C19—C20	−7.8 (2)	C1—C5—Fe1—C10	−169.41 (9)
C23—C19—C20—C21	−1.0 (2)	C4—C5—Fe1—C2	−81.81 (10)
C18—C19—C20—C21	179.35 (14)	C1—C5—Fe1—C2	37.40 (9)
C19—C20—C21—N2	−0.3 (2)	C4—C5—Fe1—C8	156.08 (9)
N2—C22—C23—C19	−0.5 (2)	C1—C5—Fe1—C8	−84.71 (11)
C20—C19—C23—C22	1.3 (2)	C4—C5—Fe1—C1	−119.21 (13)
C18—C19—C23—C22	−178.98 (14)	C2—C1—Fe1—C4	81.96 (10)
C29—C25—C26—C27	0.1 (2)	C5—C1—Fe1—C4	−37.79 (9)
Fe2—C25—C26—C27	60.38 (12)	C2—C1—Fe1—C7	−82.45 (10)
C29—C25—C26—Fe2	−60.25 (12)	C5—C1—Fe1—C7	157.79 (9)
C25—C26—C27—C28	−0.3 (2)	C2—C1—Fe1—C3	37.81 (9)
Fe2—C26—C27—C28	59.76 (13)	C5—C1—Fe1—C3	−81.95 (10)
C25—C26—C27—Fe2	−60.03 (11)	C2—C1—Fe1—C9	−168.44 (9)
C26—C27—C28—C29	0.3 (2)	C5—C1—Fe1—C9	71.81 (11)
Fe2—C27—C28—C29	59.50 (13)	C2—C1—Fe1—C6	−45.40 (18)
C26—C27—C28—Fe2	−59.18 (12)	C5—C1—Fe1—C6	−165.16 (13)
C27—C28—C29—C25	−0.2 (2)	C2—C1—Fe1—C10	153.6 (2)
Fe2—C28—C29—C25	59.25 (13)	C5—C1—Fe1—C10	33.9 (3)
C27—C28—C29—Fe2	−59.49 (13)	C5—C1—Fe1—C2	−119.75 (13)
C26—C25—C29—C28	0.1 (2)	C2—C1—Fe1—C8	−126.14 (9)
Fe2—C25—C29—C28	−59.60 (13)	C5—C1—Fe1—C8	114.11 (9)
C26—C25—C29—Fe2	59.66 (12)	C2—C1—Fe1—C5	119.75 (13)
C34—C30—C31—C32	−0.12 (17)	C29—C25—Fe2—C30	−176.60 (10)
Fe2—C30—C31—C32	59.26 (11)	C26—C25—Fe2—C30	64.75 (14)
C34—C30—C31—Fe2	−59.38 (11)	C29—C25—Fe2—C26	118.65 (15)
C30—C31—C32—C33	0.18 (18)	C29—C25—Fe2—C27	80.07 (12)
Fe2—C31—C32—C33	58.98 (11)	C26—C25—Fe2—C27	−38.57 (12)
C30—C31—C32—Fe2	−58.80 (11)	C29—C25—Fe2—C28	37.12 (11)
C31—C32—C33—C34	−0.17 (18)	C26—C25—Fe2—C28	−81.53 (12)
Fe2—C32—C33—C34	58.77 (10)	C26—C25—Fe2—C29	−118.65 (15)
C31—C32—C33—Fe2	−58.94 (11)	C29—C25—Fe2—C31	−136.53 (10)
C31—C30—C34—C33	0.01 (17)	C26—C25—Fe2—C31	104.82 (11)
Fe2—C30—C34—C33	−59.66 (10)	C29—C25—Fe2—C33	−54.99 (16)
C31—C30—C34—C35	179.96 (14)	C26—C25—Fe2—C33	−173.64 (12)
Fe2—C30—C34—C35	120.29 (15)	C29—C25—Fe2—C32	−92.54 (12)
C31—C30—C34—Fe2	59.67 (11)	C26—C25—Fe2—C32	148.81 (11)
C32—C33—C34—C30	0.10 (17)	C31—C30—Fe2—C25	67.72 (12)
Fe2—C33—C34—C30	59.34 (10)	C34—C30—Fe2—C25	−172.89 (9)
C32—C33—C34—C35	−179.85 (14)	C31—C30—Fe2—C26	106.69 (10)
Fe2—C33—C34—C35	−120.61 (14)	C34—C30—Fe2—C26	−133.92 (9)
C32—C33—C34—Fe2	−59.25 (11)	C31—C30—Fe2—C34	−119.39 (12)
C30—C34—C35—C40	168.91 (15)	C31—C30—Fe2—C27	150.28 (9)
C33—C34—C35—C40	−11.2 (2)	C34—C30—Fe2—C27	−90.33 (10)
Fe2—C34—C35—C40	−100.58 (14)	C31—C30—Fe2—C28	−175.46 (12)
C30—C34—C35—C36	−8.7 (2)	C34—C30—Fe2—C28	−56.07 (15)
C33—C34—C35—C36	171.26 (14)	C34—C30—Fe2—C31	119.39 (12)
Fe2—C34—C35—C36	81.82 (17)	C31—C30—Fe2—C33	−81.04 (9)
C40—C35—C36—C37	−0.8 (2)	C34—C30—Fe2—C33	38.35 (8)
C34—C35—C36—C37	176.83 (14)	C31—C30—Fe2—C32	−37.35 (8)
C35—C36—C37—C38	0.5 (2)	C34—C30—Fe2—C32	82.04 (9)
C36—C37—C38—C39	−0.1 (2)	C27—C26—Fe2—C25	−117.50 (17)
C36—C37—C38—N3	−179.95 (14)	C27—C26—Fe2—C30	105.73 (12)
C37—C38—C39—C40	0.0 (2)	C25—C26—Fe2—C30	−136.77 (11)
N3—C38—C39—C40	179.89 (13)	C27—C26—Fe2—C34	65.67 (14)
C38—C39—C40—C35	−0.3 (2)	C25—C26—Fe2—C34	−176.83 (11)
C36—C35—C40—C39	0.7 (2)	C25—C26—Fe2—C27	117.50 (17)
C34—C35—C40—C39	−176.97 (14)	C27—C26—Fe2—C28	−36.93 (12)
N3—C41—C42—C43	−174.09 (15)	C25—C26—Fe2—C28	80.57 (13)
N3—C41—C42—C46	5.2 (2)	C27—C26—Fe2—C29	−80.00 (12)
C46—C42—C43—C44	−2.5 (2)	C25—C26—Fe2—C29	37.50 (12)
C41—C42—C43—C44	176.75 (14)	C27—C26—Fe2—C31	149.22 (11)
C42—C43—C44—N4	2.1 (2)	C25—C26—Fe2—C31	−93.28 (12)
N4—C45—C46—C42	1.5 (2)	C27—C26—Fe2—C32	−174.82 (13)
C43—C42—C46—C45	0.9 (2)	C25—C26—Fe2—C32	−57.32 (18)
C41—C42—C46—C45	−178.41 (15)	C33—C34—Fe2—C30	118.40 (12)
C19—C18—N1—C14	−178.86 (13)	C35—C34—Fe2—C30	−122.16 (16)
C15—C14—N1—C18	−167.87 (14)	C30—C34—Fe2—C26	67.64 (12)
C13—C14—N1—C18	13.4 (2)	C33—C34—Fe2—C26	−173.96 (10)
C23—C22—N2—C21	−0.8 (2)	C35—C34—Fe2—C26	−54.52 (16)
C20—C21—N2—C22	1.2 (2)	C30—C34—Fe2—C27	106.41 (10)
C42—C41—N3—C38	−179.76 (14)	C33—C34—Fe2—C27	−135.20 (10)
C39—C38—N3—C41	167.57 (14)	C35—C34—Fe2—C27	−15.75 (15)
C37—C38—N3—C41	−12.6 (2)	C30—C34—Fe2—C28	148.60 (10)
C43—C44—N4—C45	0.2 (2)	C33—C34—Fe2—C28	−93.00 (11)
C46—C45—N4—C44	−2.0 (2)	C35—C34—Fe2—C28	26.44 (15)
C5—C4—Fe1—C7	163.5 (2)	C30—C34—Fe2—C29	−177.47 (11)
C3—C4—Fe1—C7	44.4 (3)	C33—C34—Fe2—C29	−59.07 (14)
C5—C4—Fe1—C3	119.04 (13)	C35—C34—Fe2—C29	60.37 (17)
C5—C4—Fe1—C9	−84.63 (11)	C30—C34—Fe2—C31	−37.46 (8)
C3—C4—Fe1—C9	156.34 (9)	C33—C34—Fe2—C31	80.93 (9)
C5—C4—Fe1—C6	−167.50 (10)	C35—C34—Fe2—C31	−159.62 (14)
C3—C4—Fe1—C6	73.47 (12)	C30—C34—Fe2—C33	−118.40 (12)
C5—C4—Fe1—C10	−127.10 (10)	C35—C34—Fe2—C33	119.44 (16)
C3—C4—Fe1—C10	113.87 (10)	C30—C34—Fe2—C32	−81.12 (9)
C5—C4—Fe1—C2	81.41 (10)	C33—C34—Fe2—C32	37.28 (9)
C3—C4—Fe1—C2	−37.63 (9)	C35—C34—Fe2—C32	156.72 (14)
C5—C4—Fe1—C8	−52.12 (17)	C28—C27—Fe2—C25	−80.39 (12)
C3—C4—Fe1—C8	−171.16 (12)	C26—C27—Fe2—C25	38.96 (12)
C3—C4—Fe1—C5	−119.04 (13)	C28—C27—Fe2—C30	149.55 (10)
C5—C4—Fe1—C1	37.62 (9)	C26—C27—Fe2—C30	−91.10 (12)
C3—C4—Fe1—C1	−81.42 (10)	C28—C27—Fe2—C26	−119.35 (16)
C6—C7—Fe1—C4	37.3 (3)	C28—C27—Fe2—C34	105.65 (11)
C8—C7—Fe1—C4	156.7 (2)	C26—C27—Fe2—C34	−135.00 (11)
C6—C7—Fe1—C3	71.97 (12)	C26—C27—Fe2—C28	119.35 (16)
C8—C7—Fe1—C3	−168.64 (9)	C28—C27—Fe2—C29	−36.94 (11)
C6—C7—Fe1—C9	−81.47 (11)	C26—C27—Fe2—C29	82.41 (12)
C8—C7—Fe1—C9	37.93 (9)	C28—C27—Fe2—C31	−175.25 (11)
C8—C7—Fe1—C6	119.40 (14)	C26—C27—Fe2—C31	−55.90 (16)
C6—C7—Fe1—C10	−37.46 (10)	C28—C27—Fe2—C33	66.46 (14)
C8—C7—Fe1—C10	81.94 (10)	C26—C27—Fe2—C33	−174.19 (11)
C6—C7—Fe1—C2	113.50 (11)	C27—C28—Fe2—C25	82.62 (12)
C8—C7—Fe1—C2	−127.10 (9)	C29—C28—Fe2—C25	−37.39 (11)
C6—C7—Fe1—C8	−119.40 (14)	C27—C28—Fe2—C30	−53.52 (16)
C6—C7—Fe1—C5	−168.49 (15)	C29—C28—Fe2—C30	−173.53 (11)
C8—C7—Fe1—C5	−49.1 (2)	C27—C28—Fe2—C26	37.93 (11)
C6—C7—Fe1—C1	156.28 (10)	C29—C28—Fe2—C26	−82.08 (12)
C8—C7—Fe1—C1	−84.32 (11)	C27—C28—Fe2—C34	−90.21 (11)
C2—C3—Fe1—C4	−119.29 (12)	C29—C28—Fe2—C34	149.78 (10)
C2—C3—Fe1—C7	75.06 (11)	C29—C28—Fe2—C27	−120.01 (15)
C4—C3—Fe1—C7	−165.65 (9)	C27—C28—Fe2—C29	120.01 (15)
C2—C3—Fe1—C9	−168.87 (12)	C27—C28—Fe2—C33	−134.44 (10)
C4—C3—Fe1—C9	−49.59 (16)	C29—C28—Fe2—C33	105.55 (11)
C2—C3—Fe1—C6	115.43 (10)	C27—C28—Fe2—C32	−173.41 (10)
C4—C3—Fe1—C6	−125.29 (10)	C29—C28—Fe2—C32	66.58 (14)
C2—C3—Fe1—C10	158.00 (9)	C28—C29—Fe2—C25	119.48 (15)
C4—C3—Fe1—C10	−82.71 (10)	C28—C29—Fe2—C26	80.99 (12)
C4—C3—Fe1—C2	119.29 (12)	C25—C29—Fe2—C26	−38.48 (11)
C2—C3—Fe1—C8	45.6 (2)	C28—C29—Fe2—C34	−52.92 (16)
C4—C3—Fe1—C8	164.93 (19)	C25—C29—Fe2—C34	−172.39 (10)
C2—C3—Fe1—C5	−81.48 (9)	C28—C29—Fe2—C27	36.80 (11)
C4—C3—Fe1—C5	37.81 (9)	C25—C29—Fe2—C27	−82.67 (11)
C2—C3—Fe1—C1	−37.73 (9)	C25—C29—Fe2—C28	−119.48 (15)
C4—C3—Fe1—C1	81.55 (9)	C28—C29—Fe2—C31	−174.97 (11)
C10—C9—Fe1—C4	−82.08 (10)	C25—C29—Fe2—C31	65.55 (13)
C8—C9—Fe1—C4	158.67 (8)	C28—C29—Fe2—C33	−91.36 (11)
C10—C9—Fe1—C7	81.44 (9)	C25—C29—Fe2—C33	149.16 (10)
C8—C9—Fe1—C7	−37.81 (8)	C28—C29—Fe2—C32	−135.32 (11)
C10—C9—Fe1—C3	−47.65 (16)	C25—C29—Fe2—C32	105.20 (11)
C8—C9—Fe1—C3	−166.89 (11)	C30—C31—Fe2—C25	−134.90 (10)
C10—C9—Fe1—C6	37.59 (9)	C32—C31—Fe2—C25	105.31 (10)
C8—C9—Fe1—C6	−81.66 (9)	C32—C31—Fe2—C30	−119.79 (12)
C8—C9—Fe1—C10	−119.25 (12)	C30—C31—Fe2—C26	−90.33 (11)
C10—C9—Fe1—C2	162.8 (2)	C32—C31—Fe2—C26	149.88 (10)
C8—C9—Fe1—C2	43.6 (3)	C30—C31—Fe2—C34	37.95 (8)
C10—C9—Fe1—C8	119.25 (12)	C32—C31—Fe2—C34	−81.84 (9)
C10—C9—Fe1—C5	−125.08 (9)	C30—C31—Fe2—C27	−53.73 (14)
C8—C9—Fe1—C5	115.67 (9)	C32—C31—Fe2—C27	−173.53 (11)
C10—C9—Fe1—C1	−165.49 (9)	C30—C31—Fe2—C29	−173.66 (10)
C8—C9—Fe1—C1	75.26 (10)	C32—C31—Fe2—C29	66.55 (12)
C7—C6—Fe1—C4	−167.76 (9)	C30—C31—Fe2—C33	82.27 (9)
C10—C6—Fe1—C4	72.49 (11)	C32—C31—Fe2—C33	−37.52 (9)
C10—C6—Fe1—C7	−119.75 (13)	C30—C31—Fe2—C32	119.79 (12)
C7—C6—Fe1—C3	−126.86 (10)	C32—C33—Fe2—C25	−57.84 (16)
C10—C6—Fe1—C3	113.39 (9)	C34—C33—Fe2—C25	−177.53 (12)
C7—C6—Fe1—C9	82.02 (10)	C32—C33—Fe2—C30	81.30 (10)
C10—C6—Fe1—C9	−37.73 (9)	C34—C33—Fe2—C30	−38.38 (9)
C7—C6—Fe1—C10	119.75 (13)	C32—C33—Fe2—C34	119.69 (13)
C7—C6—Fe1—C2	−84.58 (11)	C32—C33—Fe2—C27	−174.50 (11)
C10—C6—Fe1—C2	155.67 (9)	C34—C33—Fe2—C27	65.81 (13)
C7—C6—Fe1—C8	37.78 (10)	C32—C33—Fe2—C28	−136.12 (11)
C10—C6—Fe1—C8	−81.97 (10)	C34—C33—Fe2—C28	104.19 (10)
C7—C6—Fe1—C5	162.8 (2)	C32—C33—Fe2—C29	−93.35 (11)
C10—C6—Fe1—C5	43.1 (3)	C34—C33—Fe2—C29	146.96 (10)
C7—C6—Fe1—C1	−52.60 (18)	C32—C33—Fe2—C31	37.54 (10)
C10—C6—Fe1—C1	−172.35 (13)	C34—C33—Fe2—C31	−82.15 (10)
C6—C10—Fe1—C4	−125.89 (9)	C34—C33—Fe2—C32	−119.69 (13)
C9—C10—Fe1—C4	114.67 (9)	C31—C32—Fe2—C25	−92.67 (11)
C6—C10—Fe1—C7	37.46 (9)	C33—C32—Fe2—C25	147.85 (11)
C9—C10—Fe1—C7	−81.98 (9)	C31—C32—Fe2—C30	37.38 (9)
C6—C10—Fe1—C3	−83.37 (10)	C33—C32—Fe2—C30	−82.10 (10)
C9—C10—Fe1—C3	157.19 (9)	C31—C32—Fe2—C26	−54.88 (17)
C6—C10—Fe1—C9	119.44 (13)	C33—C32—Fe2—C26	−174.35 (13)
C9—C10—Fe1—C6	−119.44 (13)	C31—C32—Fe2—C34	81.71 (9)
C6—C10—Fe1—C2	−51.40 (17)	C33—C32—Fe2—C34	−37.77 (9)
C9—C10—Fe1—C2	−170.85 (12)	C31—C32—Fe2—C28	−174.94 (10)
C6—C10—Fe1—C8	81.52 (9)	C33—C32—Fe2—C28	65.58 (13)
C9—C10—Fe1—C8	−37.92 (9)	C31—C32—Fe2—C29	−136.08 (10)
C6—C10—Fe1—C5	−165.95 (9)	C33—C32—Fe2—C29	104.44 (11)
C9—C10—Fe1—C5	74.60 (11)	C33—C32—Fe2—C31	−119.47 (13)
C6—C10—Fe1—C1	167.4 (2)	C31—C32—Fe2—C33	119.47 (13)
C9—C10—Fe1—C1	48.0 (3)		
